# Patients with infective endocarditis patients in the ICU: how are they?

**DOI:** 10.1186/cc10796

**Published:** 2012-03-20

**Authors:** P Fernandez Ugidos, R Gomez Lopez, P Vidal Cortes, AV Aller Fernandez, JM Lopez Perez

**Affiliations:** 1 Complejo Hospitalario Universitario Ourense, Spain; 2 Complejo Hospitalario Universitario A Coruña, Spain

## Introduction

The objective was to analyze clinical characteristics of patients with infective endocarditis (IE) requiring surgery when the disease is diagnosed.

## Methods

A retrospective study of all patients, during 5 years in a tertiary hospital in Spain, which required admission to the ICU with the diagnosis of IE (Duke criteria modified) and required surgery at the same time. We compiled demographics, clinical characteristics and complications. Data were analyzed with SPSS 17.

## Results

We had 73 patients, 79% male, mean age 65. Forty-five percent had previous heart disease. Eighty-four percent presented with fever, 56.5% general syndrome, 56.2% heart failure, 19.2% pain, and 7% coma. The duration of the clinic before diagnosis was mainly between 7 and 30 days (32%), followed by more than 30 days (27%). Less than 3 days duration represented 13%. Blood cultures were positive in 82%. The most common agent was Streptococcus (39%), followed by *Staphylococcus aureus *MS (16%), SCN (12%), Enterococcus (12.3%), *S. aureus *MR (1.4%), *Escherichia coli *(1.4%), *Pseudomonas Aeruginosa *(1.4%), Aspergillus (1.4%), and polymicrobial (1.4%). Twelve percent were negative cultures. The valve more frequently affected was aortic. In all cases TTE was carried out for diagnosis. In 69 cases TEE was performed. The principal echo findings were: vegetation (42%), new insufficiencies (26%), and also stenosis, perivalvular abscess and normal echo. Fifty-eight percent of patients had no distal emboli. Other localizations: splenic (11%), hepatic (2.7%), bones (2.7%), brain (4%), lung (5%) and more than one (11%). Forty-one percent of patients required ICU admission before surgery with an average stay of 5.6 days. A total of 31.5% suffered multiorgan failure. Antibiotics were given 17 days before surgery. In 6.8% it was not possible to give them preoperatively. Eighty-two percent of patients took combination therapy (19% four). Cephalosporins, aminoglucosids and vancomycin were the most used. Two patients died before surgery. Thirty-five percent of the interventions were urgent. In 16.4%, reoperation was necessary, mainly for bleeding, followed by prosthetic dysfunction, recurrent IE, mediastinitis and pseudoaneurysm repair. A total 56% of patients presented postoperative shock. MV was needed during 5 days (range 0 to 53). Acute renal failure post surgery was present in 58%. Other complications were secondary infection, ventricular dysfunction, atrioventricular block, stroke, perioperative MI, and liver failure. The ICU stay was 33 days (median 6). The hospital mortality was 31.5%.

## Conclusion

IE has high morbi-mortality. The subgroup of patients requiring early surgery presents the most severe disease. This corresponds with our patients: one-third of cases need urgent surgery, 56% have shock, about 60% ARF, and mortality reaches 30%.

